# Serial assessments of cardiac output and mixed venous oxygen saturation in comatose patients after out-of-hospital cardiac arrest

**DOI:** 10.1186/s13054-023-04704-2

**Published:** 2023-10-27

**Authors:** Johannes Grand, Christian Hassager, Henrik Schmidt, Simon Mølstrøm, Benjamin Nyholm, Henrik Frederiksen Høigaard, Jordi S. Dahl, Martin Meyer, Rasmus P. Beske, Laust Obling, Jesper Kjaergaard, Jacob E. Møller

**Affiliations:** 1 Department of Cardiology B, Section 2142, Copenhagen University Hospital, Rigshospitalet, Blegdamsvej 9, 2100 Copenhagen, Denmark; 2 Department of Cardiology, Copenhagen University Hospital Amager-Hvidovre, Copenhagen, Denmark; 3 Department of Cardiology, Odense University Hospital, 5000 Odense, Denmark; 4 Clinical Institute University of Southern Denmark, Odense, Denmark; 5 Department of Anesthesiology and Intensive Care, Odense University Hospital, 5000 Odense, Denmark; 6 Department of Clinical Medicine, University of Copenhagen, Copenhagen, Denmark

**Keywords:** Cardiac arrest, Vasopressors, Hemodynamic parameters, Post-cardiac arrest syndrome

## Abstract

**Aim:**

To assess the association with outcomes of cardiac index (CI) and mixed venous oxygen saturation (SvO2) in comatose patients resuscitated from out-of-hospital cardiac arrest (OHCA).

**Methods:**

In the cohort study of 789 patients included in the “BOX”-trial, 565 (77%) patients were included in this hemodynamic substudy (age 62 ± 13 years, male sex 81%). Pulmonary artery catheters were inserted shortly after ICU admission. CI and SvO2 were measured as soon as possible in the ICU and until awakening or death. The endpoints were all-cause mortality at 1 year and renal failure defined as need for renal replacement therapy.

**Results:**

First measured CI was median 1.7 (1.4–2.1) l/min/m^2^, and first measured SvO2 was median 67 (61–73) %. CI < median with SvO2 > median was present in 222 (39%), and low SvO2 with CI < median was present in 59 (11%). Spline analysis indicated that SvO2 value < 55% was associated with poor outcome. Low CI at admission was not significantly associated with mortality in multivariable analysis (*p* = 0.14). SvO2 was significantly inversely associated with mortality (hazard ratio_adjusted_: 0.91 (0.84–0.98) per 5% increase in SvO2, *p* = 0.01). SvO2 was significantly inversely associated with renal failure after adjusting for confounders (OR_adjusted_: 0.73 [0.62–0.86] per 5% increase in SvO2, *p* = 0.001). The combination of lower CI and lower SvO2 was associated with higher risk of mortality (hazard ratio_adjusted_: 1.54 (1.06–2.23) and renal failure (OR_adjusted_: 5.87 [2.34–14.73].

**Conclusion:**

First measured SvO2 after resuscitation from OHCA was inversely associated with mortality and renal failure. If SvO2 and CI were below median, the risk of poor outcomes increased significantly.

**Registration:**

The BOX-trial is registered at clinicaltrials.gov (NCT03141099, date 2017–30–04, retrospectively registered).

**Graphical abstract:**

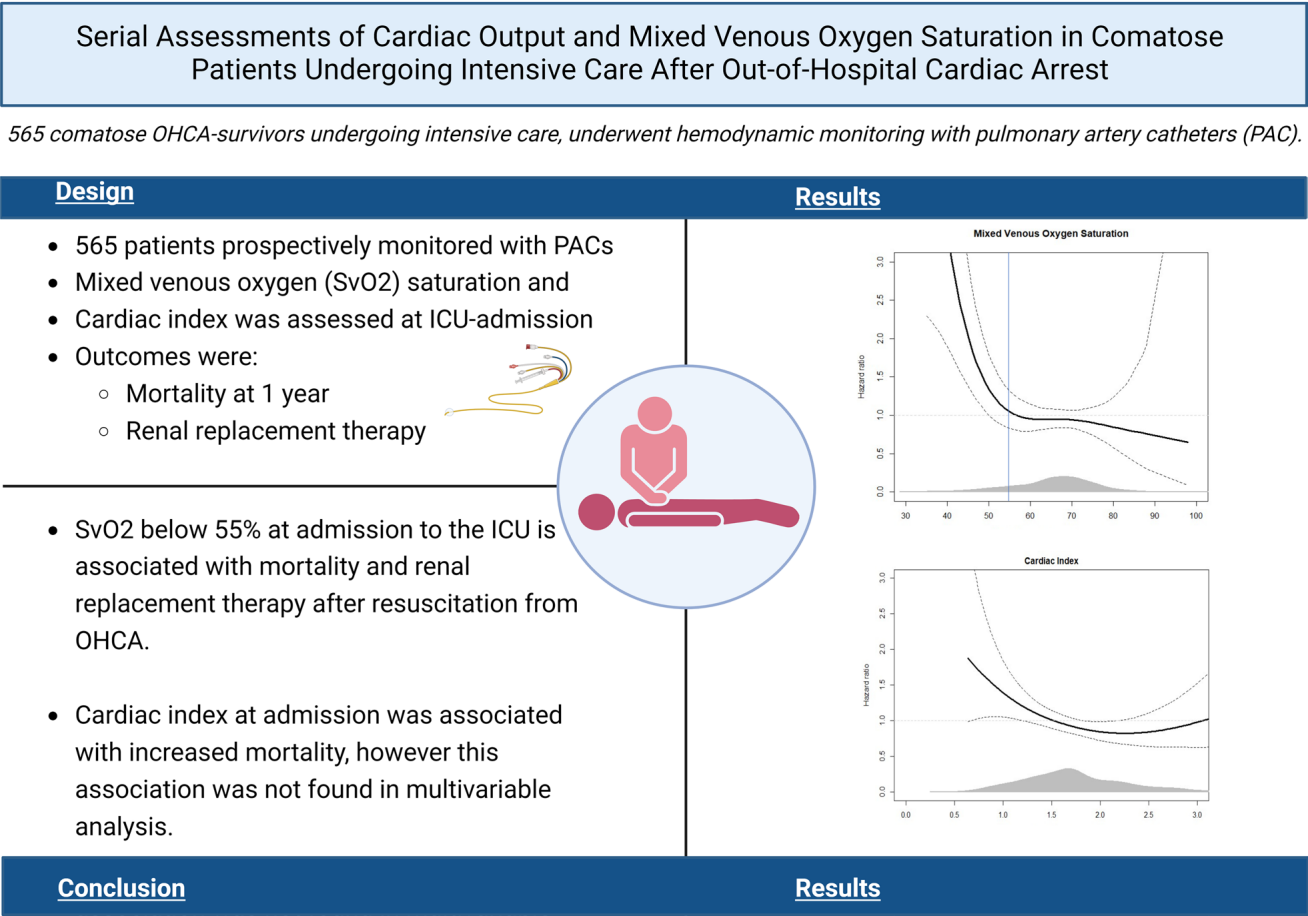

**Supplementary Information:**

The online version contains supplementary material available at 10.1186/s13054-023-04704-2.

## Take-home message

- Risk of mortality and need of renal replacement therapy increase significantly if mixed venous oxygen saturation is below 55% in the ICU after resuscitation from OHCA.
- Mixed venous SaO2 < 55% in the ICU after OHCA was associated with mortality and need of dialysis in this analysis of the BOX-trial.

## Introduction

The incidence of out-of-hospital cardiac arrest (OHCA) in Europe is 40–90 patients per 100,000 adults annually [1–3]. Resuscitated patients who remain comatose require intensive care and face an in-hospital mortality rate of 50% [4, 5]. The primary anoxic insult occurs during the cardiac arrest and subsequent compromised oxygen delivery after return of spontaneous circulation (ROSC) can potentially worsen brain injury. Impaired hemodynamics, such as hypotension, myocardial dysfunction with low cardiac output and inflammation, can contribute to inadequate oxygen delivery [6–10]. Evidence for monitoring and treating post-resuscitation hemodynamics is limited, but multiple observational studies have linked adverse outcomes to hypotension [9, 11–16]. Hypotension is treated with vasopressors, which is used frequently in post-resuscitation care [3, 17]. In the BOX-trial, a target mean arterial pressure (MAP) of 63 mmHg was compared with a target of 77 mmHg during post-resuscitation care and did not find differences in outcomes [18]. Pilot trials have found similar results [10, 19, 20]. Other hemodynamic targets than MAP include optimizing central venous pressure with fluids [3]. In case of tissue hypoperfusion, inotropic support can be initiated to increase myocardial contractility cardiac output and improve systemic perfusion [21]. Mixed venous oxygen saturation (SvO2) measured in venous blood from a catheter in the pulmonary artery (PAC) reflects a balance between systemic oxygen delivery and consumption. Low values may indicate reduced systemic oxygen delivery or increased oxygen demand, whereas high values can indicate hyperdynamic circulation. These conditions are frequent after OHCA [22–25], and therefore, SvO2 is frequently measured as part of goal-directed intensive care. Targets for hemodynamic and perfusion measures such as cardiac output and SvO2 remain undefined in post–cardiac arrest patients [17].

The aim of this study was to evaluate cardiac output and SvO2 during the intensive care phase in resuscitated comatose OHCA patients and to determine the prognostic value of these hemodynamic variables.

## Methods

### Study design, setting and patients

This study was a prespecified analyses from the BOX-trial, a randomized, controlled, multi-center study comparing two MAP targets (63 mmHg and 77 mmHg) in a double-blind intervention and comparing liberal and restrictive oxygenation targets in an open-label intervention [18, 26, 27]. Furthermore, all patients had device-based temperature control targeting 36 °C for 24 h followed by rewarming to 37 °C with 0.5 °C per hour to 37 °C. Then patients were randomly allocated toward 37 °C for either 12 or 48 h (for total intervention times of 36 and 72 h, respectively) [28]. The study took place in two Danish tertiary cardiac care centers from March 2017 to December 2021 and included 789 adult comatose survivors of out-of-hospital cardiac arrest (OHCA) of presumed cardiac origin (registered at clinicaltrials.gov (NCT03141099, date 2017–30–04, retrospectively registered). Patients were randomly assigned to MAP targets through offsetting the calibration factor in the blood pressure monitoring system as described in detail previously [29]. The inclusion and exclusion criteria for the BOX-trial are outlined in the main papers and in Additional file 1. Additional exclusion criteria for this substudy are death prior to PAC insertion, complications such as ventricular arrhythmias during the procedure or if PAC measurement such as thermodilution was not done within 2 h after admission [27]. Pre-hospital data were collected systematically according to Utstein guidelines. The study protocol, including the use of PACs for research purposes, was approved by the local Ethics Committee. Written informed consent was obtained from a legal representative and a medical doctor with no relation to the trial, and if the patient regained consciousness, informed consent was also obtained from the patient.

### Study procedures

Patients were enrolled within four hours of cardiac arrest, and blood pressure intervention was initiated immediately upon enrollment and continued until invasive arterial blood pressure monitoring was terminated. An ultrasound-guided insertion of a standard balloon-tipped pulmonary artery catheter (PAC) was performed through the internal jugular or subclavian vein and advanced to the pulmonary artery as soon as possible. Hemodynamic assessment, including thermodilution-based cardiac output measurements, was performed at time point “T0”, which was defined as the time where hemodynamic monitoring was in place and core temperature had reached the target of 36 °C. Hemodynamic variables, including central venous blood for SvO2 drawn from the PAC, were measured per protocol at T0, as well as at 6, 12, 24, 36 and 48 h thereafter. The PAC was removed either at the time of discharge from the ICU or after 72 h unless it was required for further clinical hemodynamic monitoring. The institutions' post-cardiac arrest care protocols have been previously described [18, 27] and detailed in the Additional file 1.

### Monitoring

Invasive blood pressure was measured in either the radial or brachial artery, while CVP was measured from the proximal port of the PAC. At the Copenhagen site, a 7.5F triple lumen Swan-Ganz catheter with a thermistor and balloon tip (Edwards Lifesciences, Irvine, CA) was utilized, whereas at the Odense site, a Continuous Cardiac Output (CCOmbo) PAC^®^ connected to a Vigilance II^®^ monitor (both from Edwards Lifesciences, Irvine, CA, USA) was employed with data electronically transferred to a computer at a 2-s interval. In Copenhagen, the thermodilution technique was used to assess cardiac output via an infusion of chilled isotonic glucose. Cardiac output was determined as the average of three measurements with ≤ 10% variance [30]. Our group previously investigated interobserver variation, which demonstrated low bias and high reproducibility [31]. In Odense, continuous cardiac output measurement was obtained through intermittent blood heating, with the resulting signal detected by a thermistor located near the catheter's tip [32]. Previous studies have shown excellent correlation, accuracy and precision among different methods of cardiac output measurement [31]. Hemodynamic variables were indexed to body surface area. Patients were grouped according to median CI and median SvO2. Also, patients are divided according to SvO2 above/below 55%, which was the value used to define low values during inclusion.

### Outcomes

The primary outcome is 1-year all-cause mortality.

Secondary outcomes are 1. renal failure defined as need for renal replacement therapy and 2. hemodynamic variables during 48 h of ICU admission.

### Statistical analysis

Continuous variables are presented as either mean and standard deviation (SD) or median and quartiles (q1-q3). Categorical variables are presented as count with proportions (%), and Chi-square test (or Fisher's exact test if expected counts are less than five) are used. Prespecified covariates for multivariable models are age, sex (male/female), time to ROSC, initial rhythm (shockable/non-shockable), treatment allocation (MAP 63 or 77 mmHg), left ventricular ejection fraction (LVEF) at admission, BMI, STEMI at admission and pre-existing hypertension. Hemodynamic variables were evaluated using repeated-measurements mixed models, time point and the interaction term as fixed effects. The differences between survivors and non-survivors are reported with *p* values denoted as Pgroup. Spearman’s rho (r) correlation coefficients were used to estimate associations between variables.

Skewed data are transformed either through log-transformation or square root transformation (for variables with many zero values) prior to analysis. We used the output from the mixed model to create figures, representing the geometric mean after back-transformation. Pressure variables were recorded every 10 min electronically, and the median value within that hour is used. Mortality analysis is illustrated by Kaplan–Meier plots. For illustration of the relationship between mortality and hemodynamic variables, proportional hazard model with smoothing splines was fitted. Univariable and multivariable Cox regression is used to assess association between hemodynamic variables and mortality. Results are reported as odds ratio (OR) with 95% confidence intervals (CI). The statistical analyses are performed using SAS version 9.4 and R. All tests are two-tailed, and a *p*-value of less than 0.05 is considered statistically significant.

## Results

### Patient population

**Fig. 1:**
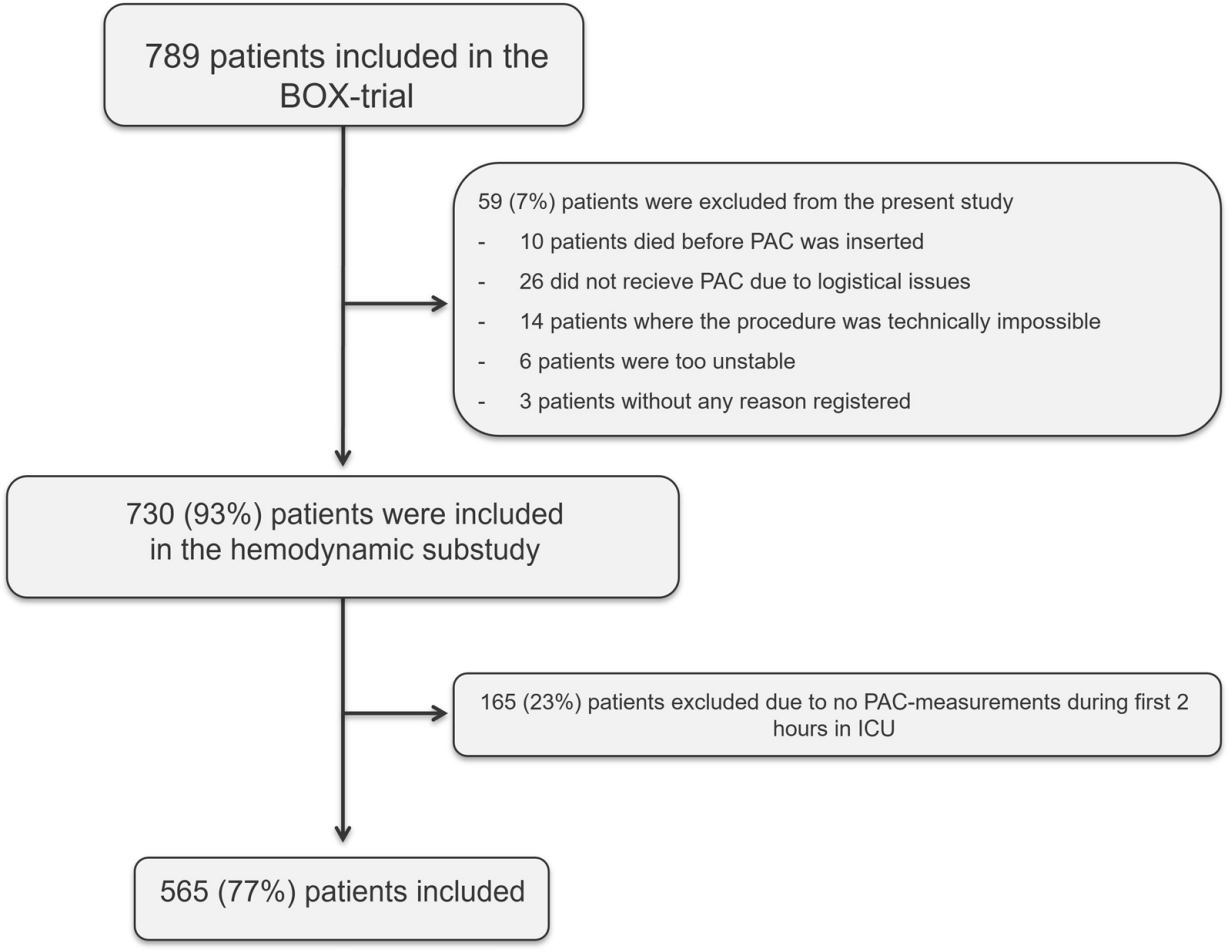
Included patients in the trial. PAC = pulmonary artery catheter

During the inclusion period, 789 were enrolled in the modified intention-to-treat population [18]. An additional 59 patients (7%) were excluded (Fig. 1) due to a failure to insert or use the PAC. Of the 730 patients with PAC placement, 165 (23%) were excluded because they did not have any PAC measurements within 2 h of ICU admission. Additional file 1: Table 1 shows demographics and cardiac arrest characteristics between patients with and without PAC measurements in the study. Overall, there was a significant difference regarding bystander use of automated external defibrillators, but other baseline variables of demographics and cardiac arrest characteristics were similar.

At 365-day follow-up, 248 (35%) had died. Table 1 shows baseline characteristics overall and between survivors and non-survivors included in the trial. Mean age was 62 ± 13 years, male sex was present in 81% of patients, and the median time to ROSC was 18 (q1–q3: 12–26) minutes. Non-survivors were significantly older and had lower incidence of witnessed arrest and bystander CPR, lower incidence of shockable primary rhythm, longer time to ROSC, higher lactate at admission, lower LVEF at admission and more comorbidities (Table 1).

**Table 1:** Demographic and prehospital data stratified according to survival status after 365 days

	Total population	Survivors at 365 days	Deceased at 365 days	*p*-value
	*n* = 565	*n* = 470 (65%)	*n* = 248 (35%)	
Demography				
Age—year (± SD)	62 ± 13	60 ± 14	67 ± 12	** < 0.0001**
Male gender—*n* (%)	458 (81%)	305 (82%)	153 (79%)	0.34
Randomization allocation				
MAP at 63 mmHg—*n* (%)	279 (51%)	185 (51%)	94 (49%)	0.69
PaO2 at 9–10 kPa—*n* (%)	284 (50%)	191 (51%)	93 (48%)	0.48
Cardiac arrest characteristics				
Witnessed arrest—*n* (%)	466 (85%)	314 (87%)	152 (80%)	**0.03**
Bystander CPR—*n* (%)	501 (88%)	343 (92%)	158 (81%)	** < 0.0001**
Bystander defibrillation—*n* (%)	125 (23%)	90 (24%)	35 (19%)	0.09
Shockable primary rhythm—*n* (%)	460 (84%)	312 (87%)	148 (76%)	**0.0001**
Time to ROSC—min. (Q1–Q3)	18 (12–26)	15 (10–20)	25 (17–33)	** < 0.0001**
Lactate at admission—mmol/L. (Q1-Q3)	5 (2.9–7.7)	3.9 (2.3–6.8)	6.0 (4.1–9.4)	** < 0.0001**
Acute CAG	513 (91%)	331 (89%)	182 (94%)	0.09
PCI—*n* (%)	229 (41%)	149 (40%)	80 (41%)	0.80
LVEF at hospital admission	35 ± 14	37 ± 14	34 ± 14	0.03
Pre-arrest comorbidities				
Previous AMI—*n* (%)	121 (21%)	73 (20%)	48 (25%)	0.09
Congestive heart failure—*n* (%)	105 (19%)	51 (15%)	54 (28%)	**0.004**
Hypertension—*n* (%)	272 (48%)	164 (44%)	108 (56%)	**0.009**
Previous TCI/stroke—*n* (%)	41 (7%)	24 (7%)	17 (9%)	0.28
Diabetes—*n* (%)	80 (14%)	41 (11%)	349(20%)	**0.003**
Chronic obstructive pulmonary disease—*n* (%)	46 (8%)	21 (6%)	25 (13%)	**0.01**
Chronic kidney disease—*n* (%)	24 (4%)	14 (4%)	10 (6%)	0.19
Atrial fibrillation—*n* (%)	99 (18%)	49 (13%)	50 (25%)	**0.0002**
Time intervals				
Time from arrest to ICU admission—h (q1–q3)	2.4 (1.9–3.1)	2.4 (1.9–3.1)	2.5 (1.9–3.1)	0.46
Time from arrest to PAC insertion—h (q1–q3)	3.0 (2.0–3.0)	3.0 (2.0–3.0)	3.0 (2.0–3.0)	0.63

CAG, coronary angiography; CPR, cardiopulmonary resuscitation; Q1–Q3, interquartile range; LVEF, left ventricular ejection fraction; *n*, number; PCI, percutaneous coronary intervention; ROSC, return of spontaneous circulation; SD, standard deviation; TCI, transitory cerebral ischemia; MAP, mean arterial blood pressure; PaO2, partial pressure of oxygen in arterial blood; kPa, kilo pascal. Bold indicates statistical significance which is a *p*-value below 0.05

### Hemodynamic parameters during intensive care between survivors and non-survivors

**Fig. 2:**
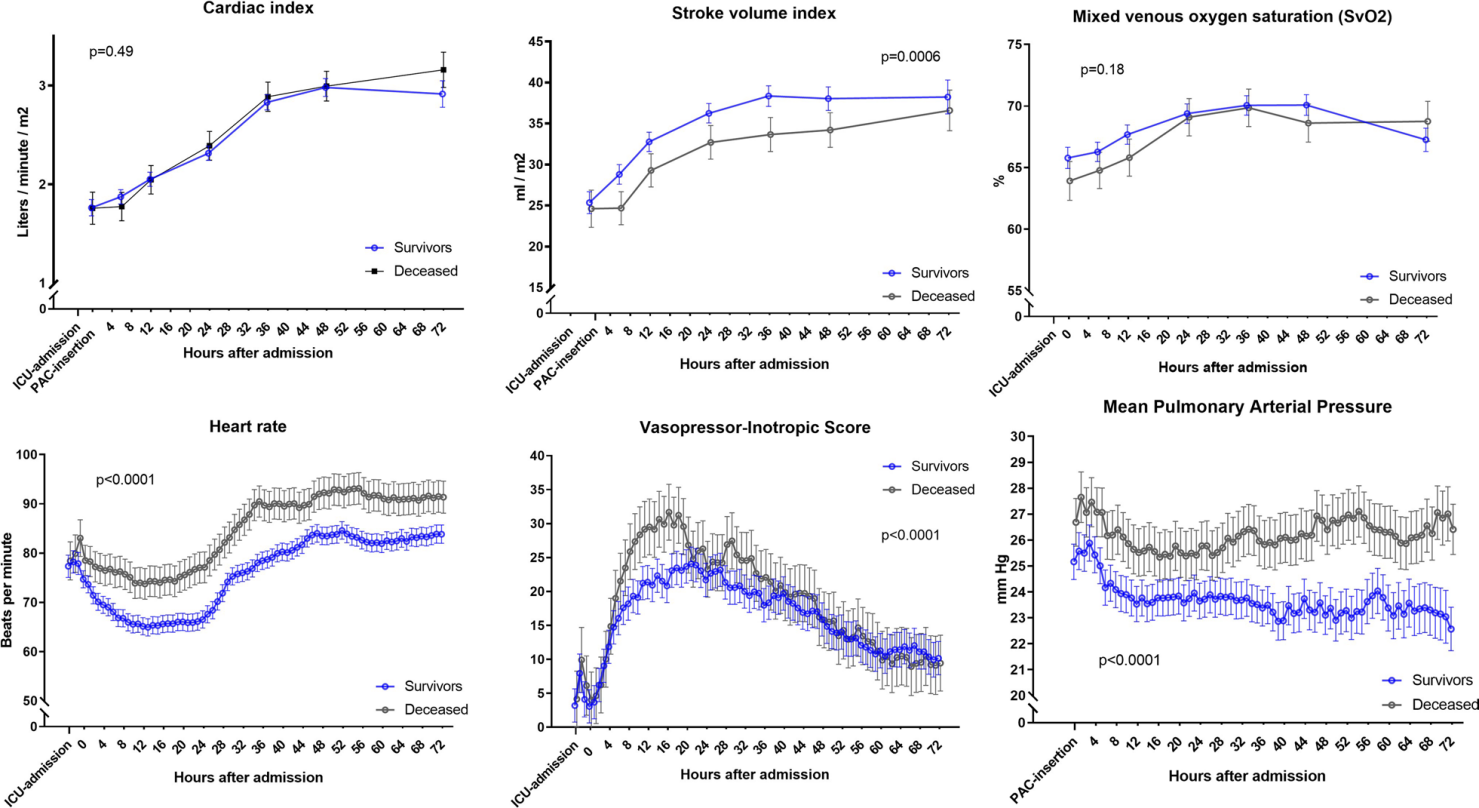
Hemodynamic status during 72 h of post-resuscitation intensive care stratified into patients surviving until 365 days and patients deceased at 365 days. *p* values indicate group difference from ICU admission until 48 h after PAC insertion. Error bars indicate 95% confidence intervals. The total amount of pharmacological circulatory support was quantified by the Vasopressor-Inotropic Score (VIS) and was calculated after the formula: Dopamine (µg/kg/min) + dobutamine (µg/kg/min) + 100 × epinephrine (µg/kg/min) + 100 × norepinephrine (µg/kg/min) + milrinone × 10 (µg/kg/min) + 50 × levosimendan (µg/kg/min) + 1000 × vasopressin (U/kg/min). The figures illustrate predicted values based on a mixed models [43]

Hemodynamic parameters at PAC insertion and values after 6, 12, 24, 36, 48 and 72 h are shown in Fig. 2. Cardiac index was not significantly different during ICU stay between 1-year survivors and non-survivors. SvO2 was significantly elevated in survivors from PAC insertion until 12 h. After 12 h the SvO2 level between survivors and non-survivors was not different. From PAC insertion until 72 h, the non-survivors had a significantly lower stroke volume index (− 4 ml/m^2^; − 5 to − 2; *p*_group_ < 0.0006, a significantly elevated mean pulmonary artery pressure (2 mmHg; 1–3), *p*_group_ < 0.0001) and significantly elevated heart rate (9 beats per minute; 7–11), *p*_group_ < 0.0001). Additional file 1: Fig. 1 shows MAP and overall doses of dopamine and noradrenaline at PAC insertion and values after 6, 12, 24, 36, 48 and 72 h.

Mean arterial pressure was lower in non-survivors (*p*_group_ < 0.0001) irrespective of MAP allocation (Additional file 1: Fig. 2).

### Cardiac output and venous oxygen saturation

**Fig. 3:**
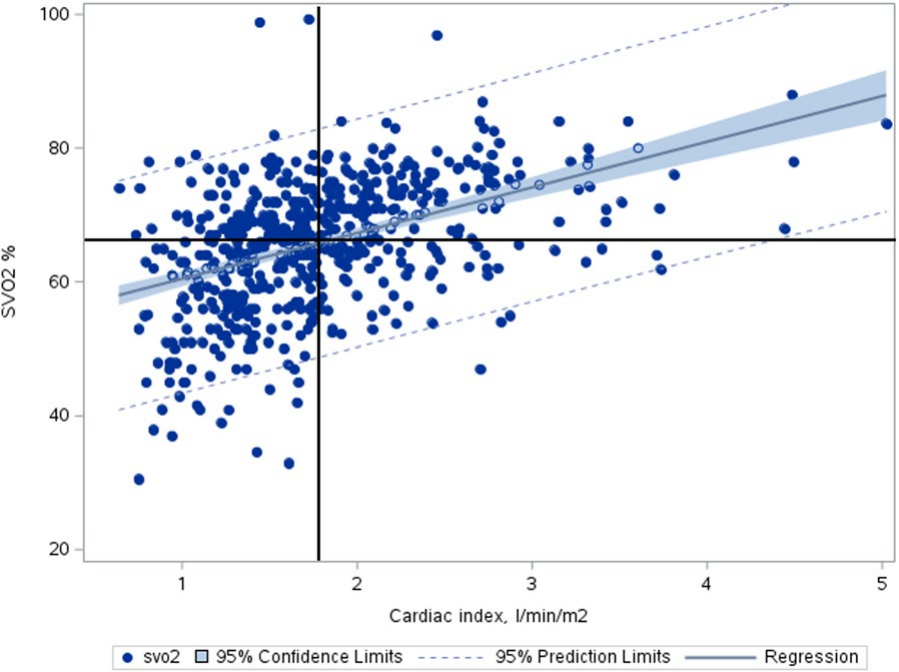
Correlation between first measured mixed venous saturation and first measured cardiac index in all patients with regression line (solid line), 95% confidence limits (filled color area) and 95% prediction limits (thin solid line). Dots change color within the filled color area to improve contrast. Vertical line indicates cardiac index at the median value, and horizontal line indicates mixed venous oxygen saturation (SVO2) at the median value

CI and SvO2 were available in 565 patients. First measured cardiac index and SvO2 correlated significantly with a Pearson correlation coefficient of 0.46, *p* < 0.0001 (Fig. 3). There was no interaction between this correlation and allocated blood pressure target.

#### Mortality

First measured CI was median 1.7 (1.4–2.1) l/min/m^2^, and first measured SvO2 was median 67 (61–73) %. The combination SvO2 > median and CI < median was present in 222 (39%), SvO2 < median and CI > median was present in 13 (2%), SvO2 < median and CI < median was present in 59 (10%), and SvO2 > and CI > median was present in 271 (48%). In 119 (21%) the first measured CI was above 2.2 l/min/m^2^.

**Fig. 4:**
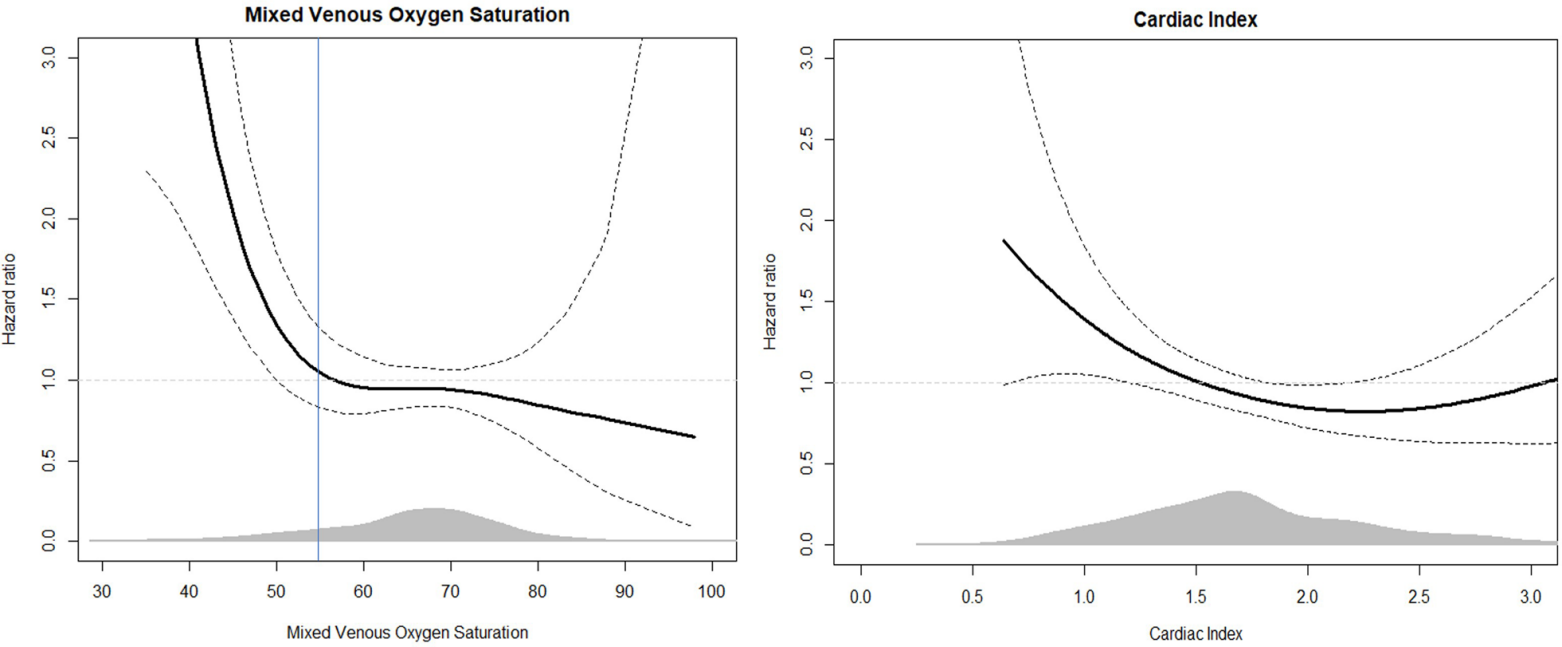
Hazard ratio of mortality as a function of first measured mixed venous oxygen saturation (left) and first measured cardiac index (right) values during intensive care after cardiac arrest. The figure is illustrated as a proportional hazard model with smoothing splines. The vertical line represents used treatment goal of > 55% mixed venous saturation

First measured CI was not significantly associated with mortality in uni- or multivariable analysis. SvO2 was associated with mortality in multivariable analysis (HR_adjusted_: 0.90 (0.84–0.98) per 5% increase in SvO2, *p* = 0.01). Spline analysis indicated that SvO2 value below 55% was associated with poor outcome (Fig. 4). Table 2 shows associations between first measured cardiac index and all-cause mortality and first measured SvO2 and all-cause mortality. CI as a continuous variable did not satisfy the assumption of linear dependence in the logistic regression model, and CI was instead analyzed in quartiles.

**Table 2:** Hazard ratios for association of cardiac index and mixed venous oxygen saturation upon insertion of pulmonary artery catheter (T0) and death from all causes at 356 days

	Hazard ratios for death							
	Cardiac Index (*n* = 565)				Mixed Venous Oxygen Saturation (*n* = 542)			
	Univariable HR (95% CL)	*p*-value	Multivariable* HR (95% CL)	*p*-value	Univariable HR (95% CL)	*p*-value	Multivariable* HR (95% CL)	*p*-value
Cardiac index/quartile 1, *n* = 142	1.58 (1.07–2.33)	0.02	1.35 (0.91–2.01)	0.14				
Cardiac index/quartile 2, *n* = 141, reference	-	-	-	-				
Cardiac index/quartile 3, *n* = 141	1.06 (0.69–1.62)	0.78	0.93 (0.61–1.44)	0.75				
Cardiac index/quartile 4, *n* = 141	1.00 (0.65–1.55)	0.99	0.78 (0.49–1.12)	0.28				
Mixed Venous Oxygen Saturation/5%					0.89 (0.83–0.96)	**0.001**	0.91 (0.84–0.98)	**0.01**
Age at arrest/5 year	1.19 (1.12–1.28)	** < 0.0001**	1.19 (1.11–1.29)	** < 0.0001**			1.19 (1.11–1.28)	** < 0.0001**
Sex, female	1.03 (0.69–1.52)	0.89	1.23 (0.86–1.75)	0.26			1.23 (0.86–1.75)	0.26
BMI	1.03 (1.01–1.06)	**0.02**	1.02 (0.99–1.06)	0.31			1.03 (0.99–1.06)	0.10
Allocated to MAP 77 mmHg	1.12 (0.83–1.50)	0.44	1.18 (0.88–1.58)	0.25			1.12 (0.84–1.49)	0.43
Allocated to liberal PaO2-target	1.12 (0.83–1.51)	0.46	1.03 (0.76–1.37)	0.86			1.05 (0.79–1.41)	0.70
Time to ROSC/min	1.03 (1.03–1.04)	** < 0.0001**	1.03 (1.03–1.04)	** < 0.0001**			1.03 (1.02–1.04)	** < 0.0001**
Shockable primary rhythm	0.58 (0.40–0.82)	**0.003**	0.59 (0.42–0.85)	**0.0034**			0.60 (0.42–0.87)	**0.0075**
STEMI	0.95 (0.70–1.29)	0.75	1.04 (0.77–1.40)	0.78			1.06 (0.79–1.42)	0.69
LVEF upon admission	0.99 (0.98–0.99)	**0.01**	1.01 (0.99–1.01)	0.80			1.00 (0.99–1.01)	0.93
Hypertension	1.43 (1.06–1.93)	**0.02**	1.12 (0.81–1.52)	0.61			1.11 (0.81–1.52)	0.44

*CL* Confidence limit, *BMI* body mass index, *MAP* mean arterial blood pressure, *PaO2* arterial partial pressure of oxygen, *CPR* cardiopulmonary resuscitation, *STEMI* ST-elevation myocardial infarction, *HR* hazard ratio, *ROSC* return of spontaneous circulation. Bold indicates statistical significance which is a *p*-value below 0.05

**Fig. 5:**
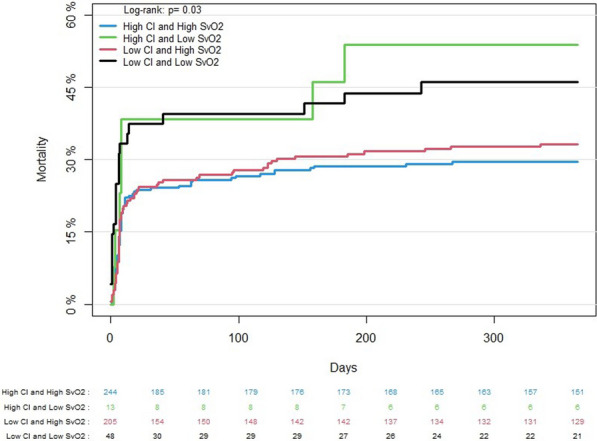
365-day mortality of study population. Patients are stratified by first measured cardiac index (> 2.2 l/min/m^2^) and SvO2 (> 55%)

Quartile of CI was not significantly associated with all-cause mortality in multivariable analysis. SvO2 > 55% was associated with lower risk of death (HR: 0.61 (0.42–0.89), *p* = 0.01. However, when adjusting for confounders, this association was no longer statistically significant (HR_adjusted_: 0.69 (0.46–1.02), *p* = 0.06). Covariates associated with mortality in multivariable model were age, time to ROSC and primary rhythm (Table 2). Patients with low SvO2 had higher mortality rates irrespective of CI (Fig. 5).

The combination of high CI and elevated lactate (CI < median and lactate > 2.5 mmol/L) was significantly associated with mortality (HR_adjusted_: 2.01 [1.29–3.13] (compared with CI > median and lactate < median), *p* = 0.002). Elevated lactate without low CI was not significantly associated with mortality (HR_adjusted_: 1.35 [0.83–2.20, *p* = 0.22]).

#### Renal replacement therapy

Additional file 1: Table 2 shows association between first measured CI and renal failure and first measured SvO2 and renal failure. CI was not associated with renal failure. However, SvO2 was associated with renal failure in multivariable analysis (OR_adjusted_: 0.73 (0.62–0.86) per 5% increase in SvO2, *p* = 0.001), Additional file 1: Fig. 3. SvO2 < median was significantly associated with renal failure (OR_adjusted_: 2.66 (1.34–5.29), p = 0.003).

The combination of CI < median and SvO2 < median was significantly associated with renal failure in univariable and multivariable analyses (OR_adjusted_: 5.87 [2.34–14.73] (compared with CI > median and SvO2 > median), *p* = 0.0001). The same trend was found for CI > median and SvO2 < median (OR_adjusted_: 3.14 (1.11–8.94)), whereas CI < median and SvO2 > median (OR_adjusted_: 1.15 [0.35–3.82] were not associated with renal failure compared to compared with CI > median and SvO2 > median.

## Discussion

This is one of the largest clinical cohorts of patients with invasive hemodynamic measurements during post-resuscitation care. We investigated the hemodynamic profile with PACs of patients resuscitated from OHCA and remaining comatose during ICU care. The main findings are that first measured cardiac index after resuscitation from OHCA despite being low in most patients was only associated with mortality and renal failure before adjusting for confounders. It seems that low cardiac index by itself does not cause hypoperfusion with adverse outcomes but is rather a marker of poor hemodynamic condition. However, lower SvO2 was associated with both mortality and renal failure, and risk seemed to increase significantly at values below 55%.

Hemodynamic monitoring is a central part of post-resuscitation intensive care of comatose patients. Almost all patients are monitored with serial blood gas analysis, invasive blood pressure and mixed or central venous oxygen saturation. In some centers, cardiac index is measured by a PAC is preferred; others use pulse index continuous cardiac output or echocardiography [33, 34]. Monitoring is used to achieve hemodynamic treatment targets using vasoactive drugs with or without inotropic effects, ventilator settings and fluid therapy [35]. However, optimal hemodynamic targets are largely based on expert consensus and unknown whether reaching specific targets improve outcome during post-resuscitation care [3]. Only a few observational studies and no randomized trials have investigated whether low cardiac index and low SvO2 after OHCA are related to clinical outcomes. These few previous studies are limited by including few selected patients from large cohorts in addition to retrospective study designs [21–23, 25, 36]. We found a significantly higher heart rate and lower stroke volume in patients with poor outcome. Overall CI was not associated with outcomes. When stratifying into quartiles, the lowest quartile of CI on admission was associated with higher mortality, but after adjusting for covariates, no quartile of CI on admission was associated with mortality.

Our group showed in a previous analysis of a small sample, that low cardiac index was not associated with poor outcome by itself [33]. Association between hemodynamic variables and outcome is likely more complex than manipulating single hemodynamic variables with drugs. In our previous study, we found that when there also were signs of hypoperfusion such as elevated lactate, low cardiac index was a marker of poor outcome [33] and we confirmed these findings in this study. That analysis in addition to many previous analyses of central hemodynamics after OHCA, was limited by a small sample size. The present study is important, since this is a large study of patients with protocolized use of PAC, allowing for additional subgroup analysis. We have expanded the previous findings and investigated the interaction between SvO2 and cardiac index and demonstrated that patients with low SvO2 had higher mortality rates irrespective of CI.

SvO2 reflects cardiovascular physiology including oxygen delivery and systemic oxygen extraction. Further, CI and SVO2 through the Fick principle are linked to oxygen consumption. If the body’s oxygen demand is low, SvO2 can in theory remain normal but cardiac output will be reduced without hypoperfusion [37, 38].

If CI is inadequate to meet oxygen demand, oxygen extraction will increase and SvO2 will reduce to maintain oxygen delivery. Oxygen extraction reflect the metabolic demands. Furthermore, peripheral factors such as adequate microcirculation and mitochondrial oxygen utilization are needed to maintain oxygen extraction. Intuitively, low SvO2 is a feature of compromised hemodynamic state only when systemic oxygen delivery is low relative to demand. Figure 3 illustrates that the hemodynamic state of CI > median and SvO2 < median was infrequent in this cohort and few patients overall had first CI values > 2.2. Low CI with adequate SvO2 is likely a reflection of low metabolic state in a patient deeply sedated, whereas low CI is associated with poor outcome only when CI is insufficient for meeting metabolic needs.

Only 21% of the cohort had CI above 2.2 l/m^2^/min which is considered normal. The relatively low CI was not associated with mortality by itself. Likely, this population due to deep sedation and mild hypothermia has lower oxygen demand and thus lower CI [39]. In a study of 95 patients undergoing temperature control, Huang et al. found that cardiac index after 12 h < 2.5 l/min/m^2^ was associated with increased mortality, which was in contrast to our findings [23]. In a study of 85 consecutive patients resuscitated from OHCA and in cardiogenic shock, Popovic et al. found significantly lower LVEF and cardiac index [40]. In 47 highly selected patients from a big cohort, Oksanen et al. reported that low cardiac index (< 1.5 l/min/m^2^) after cardiac arrest was not associated with poor outcome [24]. Torgersen et al. included 54 selected patients and showed that a higher cardiac index post-resuscitation, was weakly, but significantly associated with adverse neurological outcome [41]. The main cause of death in OHCA survivors is anoxic brain injury, and the contribution of hemodynamic status during first days in ICU to the development of irreversible brain injury is unknown. In the patient with severe irreversible anoxic brain injury, compromised hemodynamic function is likely not associated with outcome. On the other hand, it has been argued that the marginal post-anoxic brain, with some chance of recovery, is more sensitive to hemodynamic changes, and in these patients, it is particular important with a stable and “normalized” hemodynamic features [42]. This analysis found that low SvO2 is independently associated with mortality and renal failure, and future studies should evaluate whether a hemodynamic-targeted approaches could improve post-resuscitation care. If cardiac and index could be improved through a bundle of care incorporating carefully titrated fluids and/or inotropic drugs, this may result in improved outcomes. This hypothesis should be the target in a prospective trial.

### Limitations

This cohort had relatively stable hemodynamics since patients with severe hemodynamic instability with sustained cardiogenic shock was excluded Furthermore, we could not include 10 patients dying before PAC insertion, which imposes some selection of patients. This is illustrated by a relatively low mortality rate. Thus, the results cannot be extrapolated to populations with severe shock phenotypes. We used PAC measurements for cardiac index assessment and SvO2 measurements, which we consider to be the golden standard. However, 23% of patients were excluded from the analysis due to missing PAC measurements the first 2 h. Since baseline characteristics among included and excluded patients were almost similar, this likely was a consequence of logistical issues and the data can be assumed to be missing at random. Despite excluding 23% of patients, overall inclusion was high with almost all screened patients included in the main trial. Furthermore, PAC was used per protocol for all patients thus, external validity can be assumed to be high. We chose the first measured hemodynamic value to be studied in this analysis; however, analyzing SvO2 and CI at different time points during intensive care may give different results and overall differences in SvO2 during 72 h between survivors and non-survivors were small. This is an observational study, and we can report associations, which does not equal a causal relation. Furthermore, physicians were not blinded for the results of the PAC measurements and low values of SvO2, and CI may have instigated medical interventions, which may bias associations among hemodynamic variables and outcomes.

## Conclusions

A low cardiac index upon ICU admission is associated with increased mortality. However, this association disappeared when adjusting for potential confounders. Risk of mortality and renal failure increased significantly if SvO2 was low. Low SvO2 was only associated with increased risk of renal failure when CI also was low; however, patients with low SvO2 had higher mortality rates irrespective of CI.

### Supplementary Information

**Additional file 1. **Detailed information of the inclusion criteria in the BOX-trial, calculations of hemodynamic variables and supplementary figures and tables.

## Data Availability

Due to legal obligations, the supporting data are not publicly available.
