# Supplementary material for: Serial assessments of cardiac output and mixed venous oxygen saturation in comatose patients after out-of-hospital cardiac arrest
Source: Crit Care. 2023 Oct 27;27:410. doi: 10.1186/s13054-023-04704-2 (PMC10612339; doi:10.1186/s13054-023-04704-2)

Supplementary Appendix

**BOX-trial inclusion and exclusion criteria Inclusion criteria**

1. Age ≥18 years 2. OHCA of presumed cardiac cause 3. Sustained ROSC 4. Unconsciousness (GCS < 9) (patients not able to obey verbal commands) after sustained ROSC #Sustained ROSC: Sustained ROSC is when chest compressions have been not required for 20 consecutive minutes and signs of circulation persist. Exclusion criteria 1. Conscious patients (obeying verbal commands) 2. Females of childbearing potential (unless a negative HCG test can rule out pregnancy within the inclusion window) 3. In-hospital cardiac arrest (IHCA) 4. OHCA of presumed non-cardiac cause, e.g., after trauma or dissection/rupture of major artery OR cardiac arrest caused by initial hypoxia (i.e., drowning, suffocation, hanging) 5. Known bleeding diathesis (medically induced coagulopathy (e.g., warfarin, NOAC, clopidogrel) does not exclude the patient 6. Suspected or confirmed acute intracranial bleeding 7. Suspected or confirmed acute stroke 8. Unwitnessed asystole 9. Known limitations in therapy and Do Not Resuscitate-order 10. Known disease making 180 days survival unlikely 11. Known pre-arrest CPC 3 or 4 12. > 4 h (240 min) from ROSC to screening 13. Systolic blood pressure < 80 mmHg despite fluid loading/vasopressor and/or inotropic medication/ intra-aortic balloon pump/axial flow device# 14. Temperature on admission < 30 °C # If the systolic blood pressure (SBP) is recovering during the inclusion window (220 min), the patient can be included.

**Hemodynamic treatment protocol**

Patients were sedated with propofol and fentanyl to a Richmond Agitation-Sedation Scale score of -4, tracheal intubated and mechanically ventilated. Neuromuscular blocking agents were not used unless strictly necessary to reduce shivering. Hemodynamic treatment goals were 1. central venous pressure (CVP) of 10 to 15 mmHg by fluid administration, 2. Reaching the allocated MAP-target by use of vasopressors and inotropes. First-line vasopressor was noradrenaline, second line was dopamine. Target urine output was >1.5 mL/kg/hour. No general goal for targeting cardiac output was set. Emergency coronary angiography was performed for all patients presenting with ST-elevation in initial ECG in Copenhagen, and for all included patients in Odense.

**Calculations of hemodynamic variables**

Mixed central venous and arterial blood were drawn at prespecified time points and analysed for mixed venous oxygen saturation (SvO2) and lactate. The total amount of pharmacological circulatory support was quantified by the Vasopressor-Inotropic Score (VIS), and was calculated after the formula: Dopamine (µg/kg/min) + dobutamine (µg/kg/min) + 100 x epinephrine (µg/kg/min) + 100 x norepinephrine (µg/kg/min) + milrinone x 10 (µg/kg/min) + 50 x levosimendan (µg/kg/min) + 1000 x vasopressin (U/kg/min) [43].

Supplementary Table 1. Demographic and Prehospital Data stratified according to whether the patients were included or excluded from the current analysis.

|  | **Included in PAC-analysis** | **Excluded from PAC-analysis** | **p-value** |
| --- | --- | --- | --- |
|  | **n=565 (77%)** | **n=165 (23%)** |  |
|  |  |  |  |
| **Demography:** |  |  |  |
| - Age - year (±SD) | 62 ±13 | 63 ±14 | 0.27 |
| - Male gender - n (%) | 458 (81%) | 143 (80%) | 0.64 |
|  |  |  |  |
| **Allocated to blood pressure target:** |  |  |  |
| - MAP at 63 mmHg - n (%) | 279 (51%) | 89 (49%) | 0.83 |
|  |  |  |  |
| **Cardiac arrest characteristics:** |  |  |  |
| - Witnessed arrest - n (%) | 466 (85%) | 152 (85%) | 0.95 |
| - Bystander CPR - n (%) | 501 (88%) | 160 (91%) | 0.19 |
| - Bystander defibrillation - n (%) | 125 (23%) | 53 (30%) | **0.009** |
| - Shockable primary rhythm - n (%) | 460 (84%) | 156 (87%) | 0.13 |
| - Time to ROSC - min. (Q1-Q3) | 18 (12-26) | 19 (13-25) | 0.72 |
| - Lactate at admission – mmol/L (Q1-Q3) | 5 (2.9-7.7) | 4.4 (2.8-8) | 0.97 |
| - Acute CAG | 513 (91%) | 148 (94%) | 0.05 |
| - PCI - n (%) | 229 (41%) | 69 (44%) | 0.47 |
|  |  |  |  |
| **Time intervals** |  |  |  |
| - Time from arrest to ICU-admission – h (q1-q3) | 3.0 (2.0-3.0) | 3 (2.0-3.0) | 0.18 |
|  |  |  |  |

Abbreviations: CAG: coronary angiography, CPR: cardiopulmonary resuscitation, Q1-Q3: interquartile range; LVEF: left ventricular ejection fraction, n: number, PCI: percutaneous coronary intervention, ROSC: return of spontaneous circulation, SD: standard deviation, TCI: transitory cerebral ischemia, MAP: mean arterial blood pressure.

Bold values indicate statistical significance, meaning a p-value<0.05

Supplementary Table 2, odds ratios for association of cardiac index and mixed venous oxygen saturation upon insertion of pulmonary artery catheter (T0) and need for renal replacement therapy afterwards.

|  | Odds ratios for renal replacement therapy | | | | | | | |
| --- | --- | --- | --- | --- | --- | --- | --- | --- |
|  | Cardiac Index | | | | Mixed Venous Oxygen Saturation | | | |
|  | **Univariable**  **OR (95%CL)** | **p-value** | **Multivariable***  **OR (95%CL)** | **p-value** | **Univariable**  **OR (95%CL)** | **p-value** | **Multivariable***  **OR (95%CL)** | **p-value** |
|  |  |  |  |  |  |  |  |  |
| Cardiac index / L/min/m2 | 0.86 (0.54-1.37) | 0.52 | 0.73 (0.440-1.22) | 0.23 |  |  |  |  |
| Mixed Venous Oxygen Saturation, / 5% |  |  |  |  | 0.74 (0.65-0.86) | **<0.0001** | 0.73 (0.62-0.86) | **0.001** |
| Age at arrest / 5 year | 1.04 (0.93-1.16) | 0.49 | 1.01 (0.88-1.16) | 0.97 |  |  | 0.96 (0.84-1.10) | 0.56 |
| Sex, female | 0.94 (0.44-1.99) | 0.86 | 0.97 (0.44-2.16) | 0.98 |  |  | 0.73 (0.32-1.67) | 0.46 |
| BMI | 1.06 (1.01-1.12) | **0.02** | 1.06 (0.99-1.12) | 0.06 |  |  | 1.02 (0.96-1.01) | 0.52 |
| Allocated to MAP 77 mmHg | 0.94 (0.53-1.65) | 0.82 | 1.24 (0.66-2.35) | 0.50 |  |  | 1.34 (0.71-2.55) | 0.37 |
| Allocated to liberal PaO2-target | 1.57 (0.89-2.79) | 0.12 | 1.38 (0.73-2.61) | 0.23 |  |  | 1.46 (0.77-2.78) | 0.25 |
| Time to ROSC/min | 1.05 (1.04-1.07) | **<0.0001** | 1.06 (1.03-1.07) | **<0.0001** |  |  | 1.06 (1.03-1.07) | **<0.0001** |
| Shockable primary rhythm | 0.86 (0.41-1.78) | 0.68 | 0.87 (0.37-1.93) | 0.71 |  |  | 0.91 (0.38-2.15) | 0.72 |
| STEMI | 0.53 (0.29-0.98) | **0.04** | 0.58 (0.30-1.19) | 0.08 |  |  | 0.49 (0.25-0.89) | **0.02** |
| LVEF upon admission | 0.97 (0.95-0.99) | **0.001** | 0.97 (0.95-0.99) | **0.003** |  |  | 0.97 (0.96-1.01) | 0.12 |
| Hypertension | 1.60 (0.91-2.84) | 0.10 | 1.52 (0.78-2.92) | 0.23 |  |  | 1.67 (0.85-3.32) | 0.13 |
|  |  |  |  |  |  |  |  |  |

Abbreviations: CL: confidence limit, CPR: cardiopulmonary resuscitation, STEMI: ST-elevation myocardial infarction, OR: Odds ratio, ROSC: return of spontaneous circulation.

Supplementary figure 1: Blood pressure, dopamine, and noradrenaline-doses during intensive care after cardiac arrest.


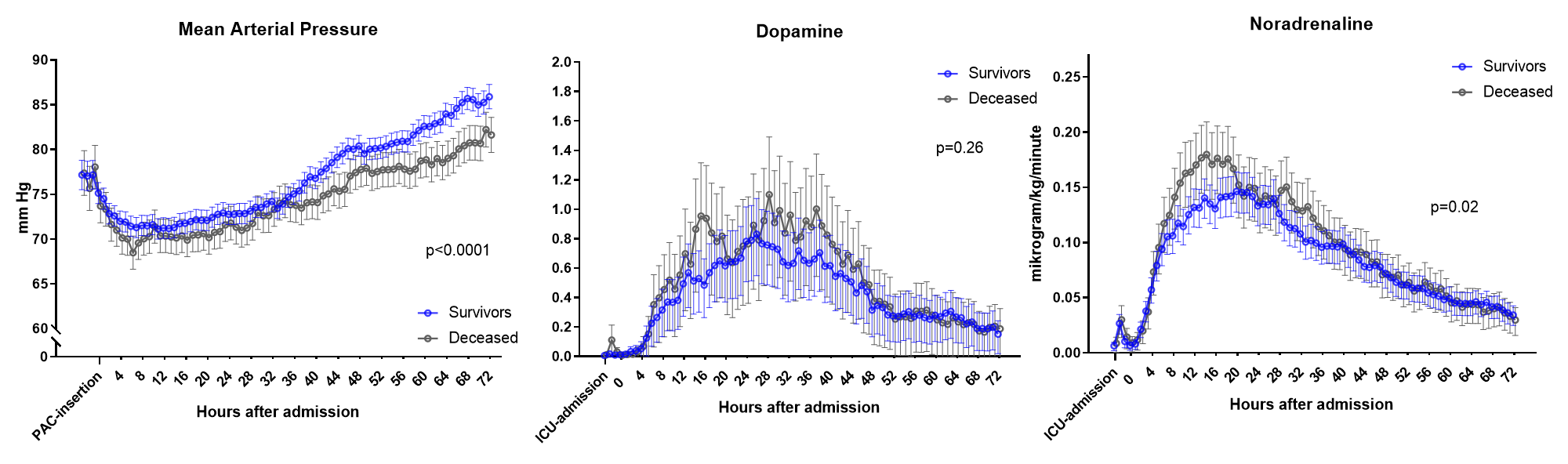


Supplementary figure 2: Blood pressure and noradrenaline-doses during intensive care after cardiac arrest divided according to allocated blood pressure target (mean arterial pressure target of 63 mmHg (MAP63) or 77 mmHg (MAP77)).


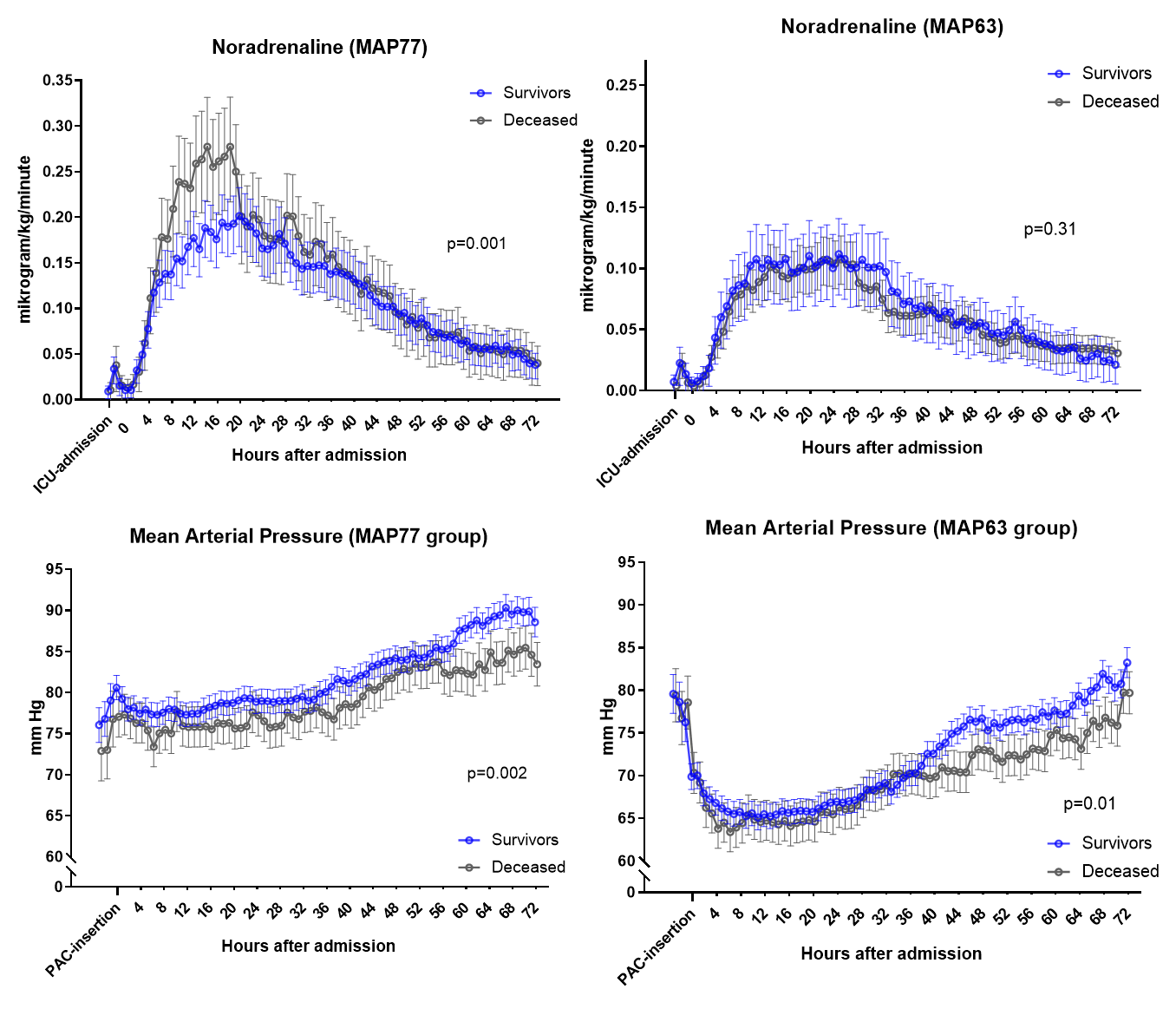


Supplementary figure 3:

Probability of renal replacement therapy at different SvO2 values at ICU-admission illustrated by a logistic regression model with cubic smoothing splines.


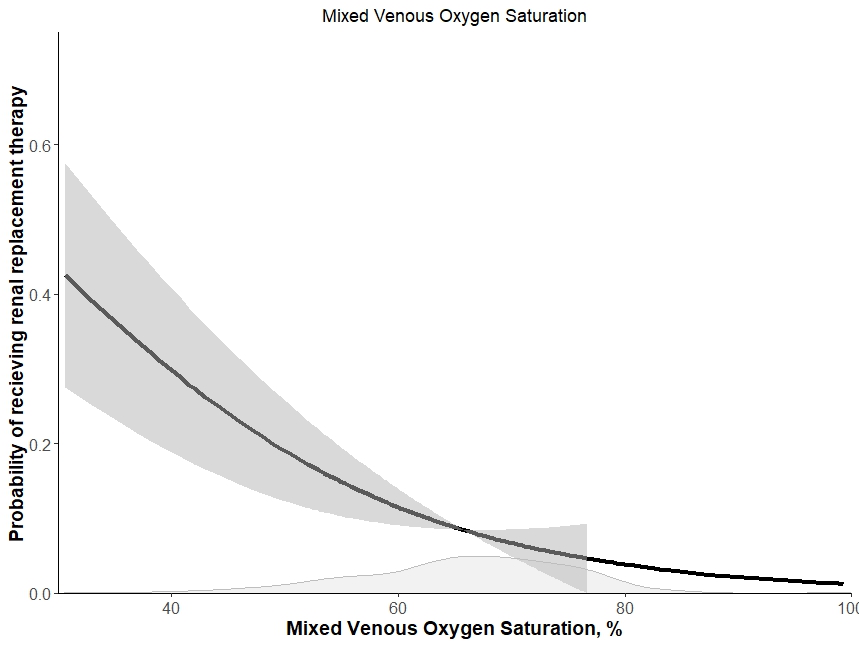

Supplement: Supplementary file 1 — Additional file 1. Detailed information of the inclusion criteria in the BOX-trial, calculations of hemodynamic variables and supplementary figures and tables. [file 13054_2023_4704_MOESM1_ESM.docx]
